# Interruption of vascular endothelial growth factor receptor 2 signaling induces a proliferative pulmonary vasculopathy and pulmonary hypertension

**DOI:** 10.1007/s00395-020-0811-5

**Published:** 2020-09-03

**Authors:** Max-Paul Winter, Smriti Sharma, Johanna Altmann, Veronika Seidl, Adelheid Panzenböck, Arman Alimohammadi, Thomas Zelniker, Bassam Redwan, Felix Nagel, David Santer, Alexander Stieglbauer, Bruno Podesser, Maria Sibilia, Thomas Helbich, Gerald Prager, Aysegül Ilhan-Mutlu, Matthias Preusser, Irene M. Lang

**Affiliations:** 1 Department of Internal Medicine II, Medical University of Vienna, Waehringer Guertel 18-20, 1090 Vienna, Austria; 2 Division of Thoracic Surgery and Lung Transplantation, Department of Cardiothoracic Surgery, University Hospital of Münster, Münster, Germany; 3 Ludwig Boltzmann Cluster for Cardiovascular Research, Center of Biomedical Research, Vienna, Austria; 4 Department of Medicine I, Institute for Cancer Research, Comprehensive Cancer Center, Medical University of Vienna, Vienna, Austria; 5 Department of Radiology, Medical University of Vienna, Vienna, Austria; 6 Department of Internal Medicine I, Medical University of Vienna, Vienna, Austria

**Keywords:** Pulmonary hypertension, VEGFR-2, FLK, *Kdr*, Murine model

## Abstract

Pulmonary arterial hypertension is a severe and progressive disease characterized by a pulmonary vascular remodeling process with expansion of collateral endothelial cells and total vessel occlusion. Endothelial cells are believed to be at the forefront of the disease process. Vascular endothelial growth factor (VEGF) and its tyrosine kinase receptor, VEGF receptor-2 (VEGFR-2), play a central role in angiogenesis, endothelial cell protection, but also in the destabilization of endothelial barrier function. Therefore, we investigated the consequences of altered VEGF signaling in an experimental model, and looked for translational correlates of this observation in patients. We performed an endothelial cell-specific conditional deletion of the *kinase insert domain protein receptor* (*kdr*) gene, coding for VEGFR-2, in C57/BL6 mice (*Kdr*^*∆end*^*)* and held them in an environmental chamber with 10% FiO_2_ or under normoxia for 6 weeks. *Kdr* knockout led to a mild PH phenotype under normoxia that worsened under hypoxia. *Kdr*^*∆end*^ mice exhibited a significant increase in pulmonary arterial wall thickness, muscularization, and VEGFR-3^+^ endothelial cells obliterating the pulmonary artery vessel lumen. We observed the same proliferative vasculopathy in our rodent model as seen in patients receiving anti-angiogenic therapy. Serum VEGF-a levels were elevated both in the experimental model and in humans receiving bevacizumab. Interrupted VEGF signaling leads to a pulmonary proliferative arteriopathy in rodents after direct ablative gene manipulation of *Kdr.* Histologically, similar vascular lesions can be observed in patients receiving anti-VEGF treatment. Our findings illustrate the importance of VEGF signaling for maintenance of pulmonary vascular patency.

**Electronic supplementary material:**

The online version of this article (10.1007/s00395-020-0811-5) contains supplementary material, which is available to authorized users.

## Introduction

Pulmonary arterial hypertension (PAH) constitutes a group of severe and progressive diseases characterized by obliteration of pulmonary arteries leading to increased pulmonary vascular resistance [5, 20]. A subsequent increase of right ventricular (RV) afterload leads to RV failure which is the leading cause of death in end-stage pulmonary hypertension (PH) [35]. Although molecular mechanisms of disease remain poorly understood [45], early changes of endothelial cells (EC) appear to be crucial in the pathogenesis of a proliferative vasculopathy that represents the key histo-pathologic finding of PAH [11, 42, 43]. For example, Tuder et al. [50] have identified tumorlets of endothelial cells obliterating medium-sized arteries and suspected deregulated EC growth to drive the vasculopathy of human PH. Endothelial cells are the building blocks of vascular networks that enable oxygen and nutrient delivery throughout a tissue, but also serve as a rich resource of factors which maintain EC integrity in an autocrine fashion. Vascular endothelial growth factor (VEGF) and its tyrosine kinase receptor, VEGF receptor-2 (VEGFR-2) play a central role in angiogenesis, endothelial cell protection, but also in the destabilization of endothelial barrier function. In vitro, specific inhibitors against VEGFR-2 [49], monoclonal antibodies directed against VEGFR-2 [26], and phenotypic knockout of VEGFR-2 are capable of inhibiting neoangiogenesis. In rodent models the inhibition of VEGF signaling by the tyrosine-kinase inhibitor Sugen5416 (SU5416) has been shown to aggravate vascular remodeling triggered by chronic hypoxia and to reproduce some of the angioproliferative features typical for advanced human PAH [44, 48]. Concomitant administration of apoptosis inhibitors could prevent the effect of growth factor inhibition, suggesting that loss of survival signals coupled with increased apoptosis lead to apoptosis-resistant ECs with abnormal growth potential [11, 48]. Because these pre-clinical models and reports from large oncology registries suggest that angiogenesis inhibitors may induce PH, we aimed to dissect the consequences of interruption of VEGFR-2 signaling [17, 29, 33]. For this purpose, we investigated hemodynamic and histological effects of direct endothelial-specific VEGFR-2 (*Kdr*) knock-out in a mouse model of chronic hypoxic pulmonary hypertension, and compared findings with pulmonary histologic changes after anti-angiogenic therapy for colorectal cancer.

## Materials and methods

### Mouse model

All animal procedures were conducted under the care and supervision of the Department of Biomedical Research of the Medical University Vienna and were approved by the Institutional Animal Care Committee and the Austrian Ministry of Science (BMBWF 66.009/0141-II/10b/2010). C57/BL6J mice with conditional *Kdr* knockout in EC (*Kdr*^*Δend*^) were used [2]. Female 8–10 weeks old *Kdr*^flox/flox^/Tie-2CreER mice served as study and Cre negative female littermates (*Kdr*^flox/flox^*/Tie-2*) served as controls. Because of lower phenotypic variability, only female mice were used in the experiments [32, 38].

### Deletion of Kdr and hypoxic breeding

Per group and timepoint 8 mice were studied***. ***Prior to experiments all mice were treated with 100 μL of tamoxifen (TX, 20 mg/mL in 10% ethanol and 90% sunflower seed oil, all Sigma Aldrich, Vienna Austria) intraperitoneally (i.p.) once daily for 5 days, followed by once a week for two consecutive weeks. *Kdr*^*flox/flox*^*/Tie-2CreER* mice and Cre-negative littermates (*Kdr*^*flox/flox*^*/Tie-2)* mice after TX treatment are labeled *Kdr*^*∆end*^ and controls, respectively. Directly after TX induction, control and *Kdr*^*∆end*^ mice constituted the groups for baseline measurements. A separate group of TX-induced animals started chronic normobaric hypoxia (10% FiO_2_) or normoxia (21% FiO_2_) in a ventilated chamber (Biospherix A chamber®, Lacona, NY, USA) for 2, 4 and 6 weeks (Supplemental Fig. 1).

**Fig. 1:**
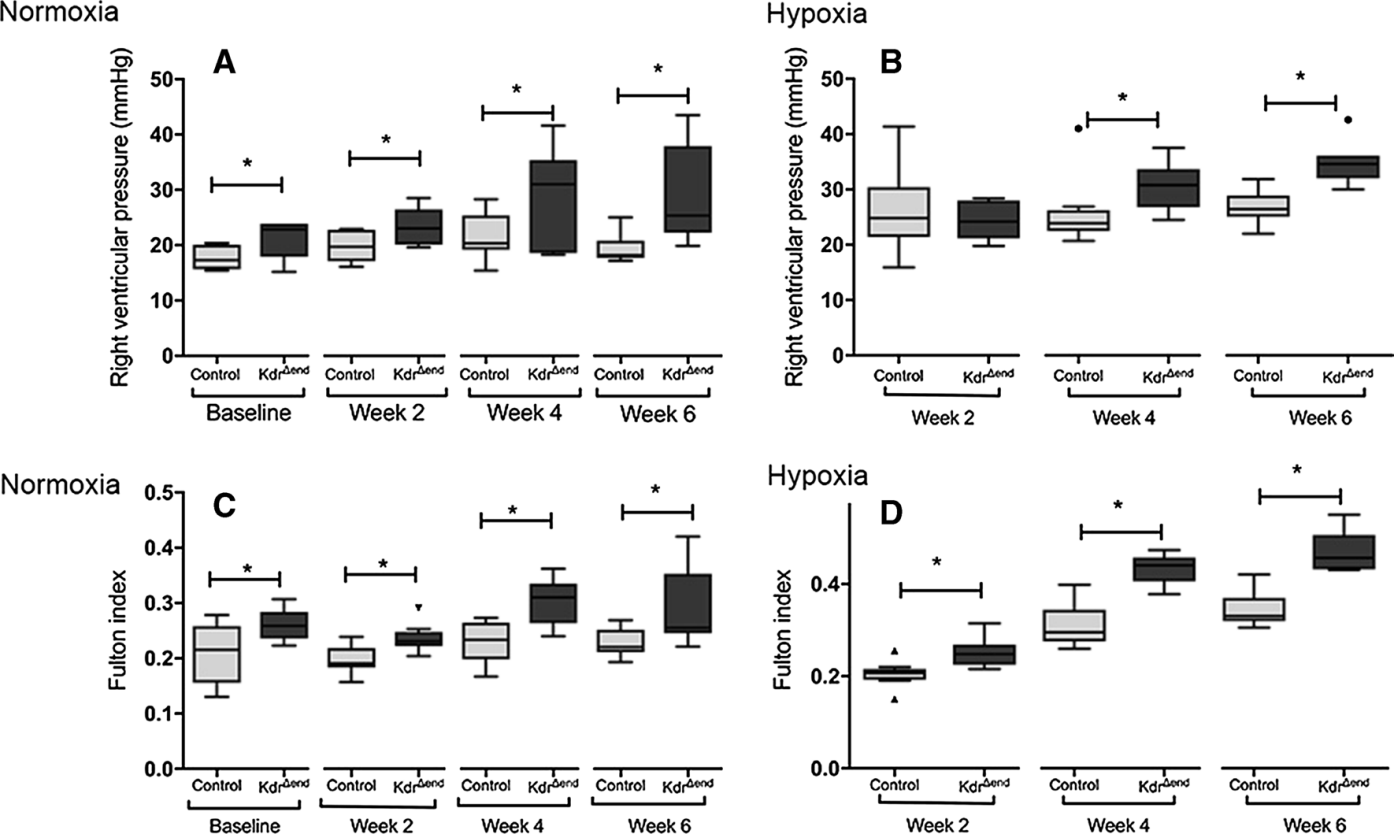
Right ventricular hemodynamic response and hypertrophy after *Kdr* knockout and hypoxic exposure. Right ventricular pressures under normoxia (*n* = 8/ group) (**a**) and hypoxia (*n* = 8/ group) (**b**), Fulton index under normoxia (*n* = 8/ group) (**c**) and hypoxia (*n* = 8/group) (**d**). Statistical differences (*p < 0.05) are determined by Student’s unpaired *t* Test. Whiskers indicate1.5xIQR

Oxygen concentration within the chamber was monitored with an oxygen analyzer (Proox 110, Biospherix, Lacona, NY, USA) and maintained by controlling nitrogen inflow rate. After the treatment period, mice were anesthetized using 1.5% isoflurane (Baxter Healthcare, Vienna, Austria) and cardiac function was assessed via magnetic resonance (MR) tomography and echocardiography. PH and vasculopathy were assessed with invasive hemodynamics, measures of right ventricular hypertrophy, histology, and molecular pathway profiling. All measurements were performed under normoxia at ambient air. Those results depicting changes after hypoxia indicate measurements taken after 6 weeks of hypoxic exposure.

### Serum biomarkers in mice

Serum VEGFa levels were determined using the Mouse VEGFa Quantikine ELISA kit (Intra-assay precision CV 4.3–8.2%, Inter-assay precision CV 5.7–8.4%, R&D Systems, Minneapolis, MN, USA). For measurements of mouse brain natriuretic peptide (BNP) concentration, Brain Natriuretic Peptide EIA Kit (Intra-assay precision CV < 10%, Inter-assay precision CV < 10%, Sigma Aldrich, Vienna, Austria) was utilized according to the manufacturer’s manual.

### Serum VEGFa in cancer patients

We prospectively collected serum samples of 34 malignant meningioma patients before and on bevacizumab therapy and measured serum VEGFa levels by a Quantikine ELISA kit (Intra-assay precision CV 4.5–6.7%, Inter-assay precision CV 6.2–8.8%, R&D Systems, Minneapolis, MN, USA). Patients gave written informed consent under a study protocol that was approved by the Ethics Committee of the Medical University of Vienna (EK 351/2005).

### Hemodynamic assessment

Mean arterial blood pressure was assessed non-invasively using a tail cuff and pulse transducer system (MLT-125 M, ADInstruments, Sydney, Australia) according to the manufacturer’s manual on the restrained mouse. Labchart 7 Pro was used for data acquisition and analysis. For assessment of right ventricular systolic pressure mice were anesthetized using 1.5% isoflurane (Baxter Healthcare, Vienna, Austria), intubated using a 23G venous cannula and mechanically ventilated with a MiniVent type 845 rodent micro-ventilator (Hugo Sachs Elektronik, March-Hugstetten, Germany). Via a bilateral anterolateral thoracotomy a direct right ventricular puncture with a 21G needle and insertion of a micro tip catheter (SPR-1000, Millar Instruments, Houston, TX, USA) were performed.

### Histology and immunohistochemistry

After hemodynamic assessment, animals were sacrificed and lung tissue was harvested for histological and molecular profiling. The heart was removed en bloc, the atria were removed, the right ventricle was dissected from the left ventricle and the interventricular septum, and the weight ratio was determined as the Fulton index. Murine lung and heart specimens were flushed with phosphate-buffered saline (PBS) through the RV, fixed in 7.5% neutral buffered formalin and embedded in paraffin. For routine examination, 3 µm tissue sections were stained with a modified trichrome stain as described previously [15]. Immunohistochemical analyses were performed utilizing the labeled streptavidin–biotin technique with a Histostain SP kit (AEC broad spectrum Kit, life technologies, Frederick, MD, USA). Cell characterization was performed using the following primary antibodies and reagents: Biotinylated Griffonia Simplicifolia Lectin I (GSL I) isolectin B4 (Vector Laboratories, Burlingame, CA, USA), anti-mouse CD45 (Abcam, Cambridge, MA, USA), anti-mouse α-smooth muscle actin (Sigma-Aldrich, Cambridge, UK), anti-proliferating cell nuclear antigen (PCNA, Abcam, Cambridge, MA, USA) and anti-active caspase 3 (Novus Biologicals, Novus Europe**,** Abingdon, UK). Slides were examined using an Olympus BX 61 microscope equipped with cellSens Dimension imaging software (Olympus, cellSens Dimension 1.11). CD45 immunoreactivity was quantified by calculating the ratio of CD45 positive to negative cells, counted per high power field (HPF, 20 × magnification). Activated caspase 3^+^, PCNA^+^ and VEGFR-3^+^ cells were counted in 5 randomly assigned HPF (magnifications 20x). Mean linear intercept (MLI) was evaluated according to Dunnill et al. [14]. Five fields of lung tissue were digitally photographed. Three suitable fields were randomly chosen per animal and counted. Areas with large bronchi were excluded from analysis. MLI was derived by overlaying a grid over each image and counting the number of times the alveolar walls intercepted the grid lines. MLI was calculated according to the equation: MLI = (N)(L)/m (*N*  is the  number of times the transverses were placed on the tissue, *L* is the  length of the transverses, and *m* is the  sum of all intercepts). As formalin fixation and paraffin embedding of mouse lungs has only a small effect on linear dimension, no correction factor was applied to MLI [4, 30].

Medial wall thickness of partially and fully muscularized arteries was determined using the formula: 2 MT × 100/ED (MT = distance between internal and external elastic laminae, and ED = external diameter) and is reported in %.

### Murine pulmonary endothelial cell isolation

Lung tissues from *Kdr*^Δend^ mice and controls were collected in sterile PBS. Tissues were minced and digested with collagenase (2 mg/ml) for 1 h at 37 °C with occasional agitation. Single cell suspensions were obtained by pipetting the samples through 70 μm and 40 μm cell strainers followed by washing with 0.1% bovine serum albumin (BSA, Sigma Aldrich, Vienna, Austria) in PBS. In case of erythrocyte contamination, cells were incubated with red cell lysis buffer and were washed again with 0.1% BSA in PBS. The cell mixture was incubated with anti-PECAM-1 (BD Biosciences, San Jose, CA, USA) coated Dynabeads (ThermoFisher, Vienna, Austria) for 30 min at room temperature with gentle rocking. The bead/endothelial cell complexes were separated on a magnetic separator by aspirating the supernatant containing contaminating cells. Total RNA was isolated using the Reliaprep RNA Miniprep System (Promega, Madison, WI, USA) following the manufacturer’s instructions.

### Immunofluorescence staining

Paraffin-embedded sections (3 µm) were deparaffinized in xylene, rehydrated through ethanol washes and rinsed in PBS. Antigen retrieval was performed by pressure cooking for 6 min in citrate buffer, pH 6.0 (target retrieval solution, Dako, Santa Clara, CA, USA) followed by a blocking step using 2.5% BSA. Sections were stained overnight at 4 °C with the following primary antibodies: rabbit anti-α-SMA, rat anti-CD31 (both Abcam, Cambridge, UK). After three washing steps, secondary antibodies conjugated with DyLight Fluor 488 and DyLight Fluor 550 (Abcam, Cambridge, UK) were applied for 1 h at room temperature; 4′,6-diamidine-2′-phenylindole dihydrochloride (DAPI, Sigma Aldrich, Vienna, Austria) was used for nuclear staining and slides were embedded in Permafluor mounting medium (ThermoFisher, Vienna, Austria). Images were taken on a Zeiss Axio observer Z1 microscope using TissueFAXS software (version 6.06.245.103, TissueGnostics, Vienna, Austria).

All histologic examinations were evaluated by an independent observer blinded for the subjects’ status and time point.

### Ink injection

Blue ink (Davidson marking system, Bradley Products, Bloomington, MNm, USA) was injected into the non-beating left ventricle after clamping the ascending aorta to distinguish arterial from venous vessels through blue demarcation of pulmonary veins (*n* = 5) [13].

### Human tissues

We analyzed lung specimens of three patients with colorectal cancer undergoing pulmonary metastasectomy under treatment with bevacizumab, a humanized anti-VEGF monoclonal antibody. Tissues were harvested and fixed according to clinical routine. Patients gave written informed consent under a study protocol that was approved by the Ethics Committee of the Medical University of Vienna (EK 274/2011).

### TissueFAXS analysis

For TissueFAXS analysis, samples were scanned at 20-fold magnification using a high-resolution microscope and TissueFAXS software (TissueGnostics, Vienna, Austria). Isolectin B4-positive and α-smooth muscle actin-positive areas were determined and quantified by Histoquest software. Large bronchi and surrounding connective tissues were excluded from the analysis.

### Real-time PCR

Total RNA was extracted from lungs using a Reliaprep™ RNA Miniprep System (Promega, Madison, WI, USA). Complementary DNA was synthesized from 2 µg of total RNA by reverse transcription (Promega, GoScript™ Reverse Transcriptase). Quantitative fluorogenic real-time PCR was performed on an ABI PRISM 7500 Sequence Detector (Applied Biosystems, Foster City, CA, USA). Specific TaqMan primers and probes for *Kdr* (ID: Mm00440099_m1), *Bone morphogenic protein 2 *(*Bmp2)* (ID:Mm01340178_m1), *Bone morphogenic protein receptor 2* (*Bmpr-2*) (ID: Mm00432134_m1), *cadherin 5 *(*Cdh5)* (ID: Mm00486938_m1) and *Complement component 1 q *(*C1q)* (ID:Mm00432142_m1) were used (TaqMan Gene Expression Assays, Applied Biosystems). Messenger RNA (mRNA) expression levels of target genes were normalized to endogenous eukaryotic 18S ribosomal RNA levels by the ΔΔCT method. In a second step normalized gene expression levels were used for pairwise comparison between the different groups.

### Echocardiographic measurements

A Vevo2100 imaging system (VisualSonics Inc, Toronto, Canada) with a MS400 ultrasound probe was used for echocardiographic assessment. For visualization of the pulmonary artery (PA) outflow tract the MS400 probe was placed in a parasternal long axis position, and pulsed-wave Doppler mode was used to visualize and blood flow dynamics through the pulmonary valve to calculate the ratio of pulmonary acceleration time (PAT) to total pulmonary ejection time (PET). Three cardiac cycle measurements were used to average the ratio of PAT/PET, which is negatively correlated with PA pressure. Left ventricular function was estimated by cardiac output (CO) measurements, as described previously [7, 11]. An independent observer blinded to the experimental groups performed the echocardiographic measurements.

### Cardiac magnetic resonance tomography

Magnetic Resonance Tomography (MRT) was performed on a 9.4 T Biospec 94/30 USR system (Bruker Biospin, Ettlingen, Germany). A gradient insert with inner diameter of 116 mm was used. The maximal achievable gradient strength was 667mT/m. For radiofrequency excitation a transmitter volume resonator with an inner diameter of 86 mm was used, for image acquisition a dedicated mouse heart coil array with four elements was used. Mice were preanesthetized with isoflurane and positioned on a heated mouse bed. Anesthesia was maintained with 1.5–2% isoflurane via a face mask. A prospective ECG-gated cine gradient echo-based flow compensated MR sequence, which is implemented in the in-built software ParaVision 6.0 (Bruker Biospin, Ettlingen, Germany) was acquired to visualize cardiac function. A mean of ten consecutive axial slices along the long axis from the apex to the base of the heart were acquired. The following imaging acquisition parameters were used: time of echo = 2.4 ms, time of repetition = 8 ms, Averages = 6, field of view = 25 mm × 25 mm, slice thickness = 0.8 mm, flip angle = 15°, partial Fourier Transformation = 1.45, measured matrix = 132 × 192, visualized matrix = 192 × 192, 18 movie frames. An independent observer blinded to the experimental groups performed all MR measurements and another blinded observer performed the post processing of the recorded images.

### Post processing

Left ventricular function was assessed using Segment-Software for Quantitative Medical Image Analysis (Segment Software, v1.8 R1172; Medviso AB, Lund, Sweden). The cine sequence was used to determine end-systole and end-diastole. End-systolic and end-diastolic volumes (ml) were measured by manual feature-tracking of the right and left ventricle on each axial slice representing a level along the long axis excluding the papillary muscles. Ejection fraction (%) and stroke volume were calculated automatically. Cardiac output was determined as stroke volume multiplied by heart rate.

### Statistical analyses

Normal distributions of data were assessed using the Shapiro–Wilk test. The significance of intergroup differences was assessed by independent samples Student *t* test and Mann–Whitney *U* test for comparison between groups. SPSS 23.0 (IBM corp., Chicago, IL, USA) and GraphPad Prism 6 (GraphPad Software Inc., USA) were applied for statistical analysis. All results are expressed either as means ± standard error of the mean (SEM) or standard deviation (SD) or median and interquartile range (IQR). All statistical analyses of the murine model refer to comparison of *Kdr*^*Δend*^ mice to the respective control littermates, either under normoxia or hypoxia.

## Results

### Characterization of *Kdr*^∆end^ mice

*Kdr*^*flox/flox*^/*Tie-2CreER* (= *Kdr*^*∆end*^) (*n* = 8) and control mice (*n* = 8) were injected with TX followed by normoxic or hypoxic housing. All mice survived until pre-specified sacrifice and no gross anatomic differences were observed between *Kdr*^*∆end*^ mice and littermate controls. Physiologic measurements at study completion are presented in Table 1. Both under normoxia and hypoxia the study groups were indistinguishable with respect to body weight, heart weight and heart-to-body weight ratio. Mice under hypoxia showed a significant increase in hematocrit, but there was no significant difference between *Kdr*^*∆end*^ and control mice. To assess systemic cardiovascular response to *Kdr* knockout, systolic arterial blood pressure was measured in unanesthetized mice at baseline and after hypoxic housing. Systolic arterial blood pressure did not differ between experimental groups regardless of *Kdr* status or hypoxic exposure (Table 1).

**Table 1:** Mouse characteristics and hemodynamics

	Baseline			6 weeks Hypoxia		
	*Kdr*^*∆end*^	Control	*p *value	*Kdr*^*∆end*^	Control	*p* value
Bodyweight (g)	26.3 ± 1.8	25.5 ± 2.2	*n.s*	25.6 ± 1.5	23.7 ± 2.1	*n.s*
Heart weight (mg)	130 ± 10	120 ± 20	*n.s*	140 ± 10	130 ± 20	*n.s*
Heart-to-Body weight ratio (mg/g)	5.1 ± 0.3	4.8 ± 0.5	*n.s*	5.5 ± 0.30	5.7 ± 0.60	*n.s*
Hematocrit (%)	38.1 ± 1.8	38.7 ± 3.9	*n.s*	51.7 ± 3.5	53.0 ± 4.3	*n.s*
Leukocyte count (G/L)	4.0 ± 0.6	4.9 ± 1.4	*n.s*	5.6 ± 0.4	5.2 ± 0.9	*n.s*
Systemic systolic pressure (mmHg)	87.2 ± 6.9	87.6 ± 8.6	*n.s*	91.6 ± 16.0	91.4 ± 12.0	*n.s*
RVSP (mmHg)	20.7 ± 3.9	17.8 ± 2.2	< 0.05	34.8 ± 4.1	26.7 ± 2.8	< 0.001

*g* gram, *G/L* giga per liter, *mmHg* millimeter mercury, *RVSP* right ventricular pressure

### Conditional Kdr knockout leads to increased RV pressures and a pulmonary vasculopathy

**Fig. 2:**
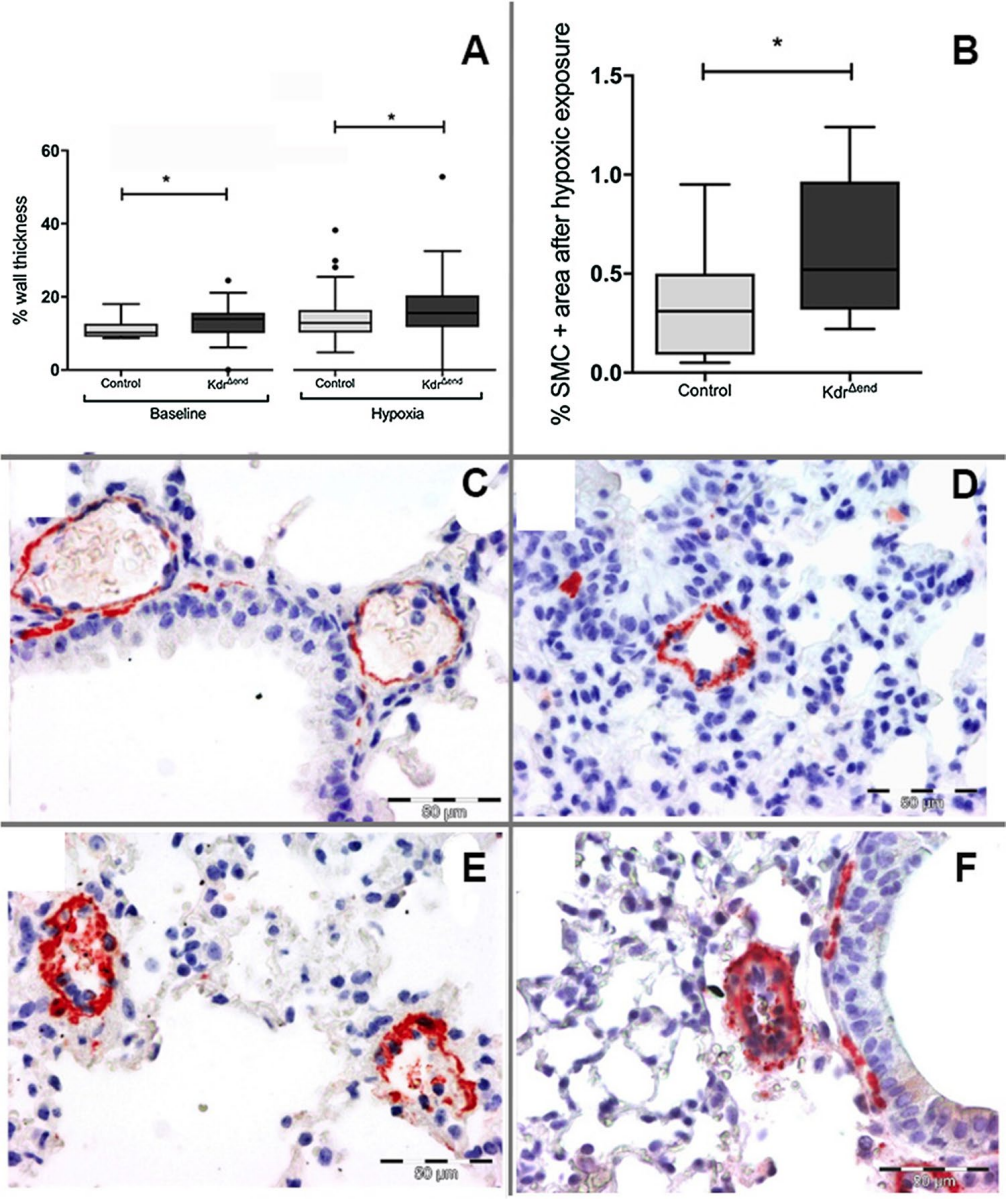
Pulmonary vascular remodeling. Pulmonary vessel wall thickness (%) of *Kdr*^*∆end*^ (*n* = 8) and controls (*n* = 8) (**a**), α-SMC^+^ area measured by TissueFAXS (*n* = 8/ group) (**b**). Control mice after induction showed no vascular abnormalities (**c**), with a mild-moderate increase in media wall thickness after hypoxic breeding (**d**), medial hypertrophy, neointimal thickening in *Kdr*^*∆end*^ mice (**e**, **f**). Statistical differences (*p < 0.05) are determined by Student’s unpaired *t* Test or Mann–Whitney *U* test as appropriate. Whiskers indicate1.5xIQR

After conditional knockout, mice showed a steady increase in RV systolic pressure (RVSP) under both normoxic and hypoxic conditions. In contrast to the SUGEN model [11], *Kdr*^*∆end*^ mice (*n* = 8) exhibited a mild PH phenotype after completion of a 3 weeks’ course of TX induction even in the absence of chronic hypoxic exposure, with significant increases of RVSP and Fulton index (Fig. 1a, b; Table 1, Supplemental Table 1). This phenotype was more severe after chronic exposure to hypoxia, with a significant increase in RVSP after 6 weeks (34.8 ± 4.1 mmHg vs. 26.7 ± 2.8 mmHg in control littermates, *p* < 0.001) (Fig. 1b, d). EC-specific conditional deletion of VEGFR-2 did not lead to development of emphysema as depicted by the mean linear intercept as a surrogate for alveolar enlargement. There was no significant difference in MLI between *Kdr*^*∆end*^ mice (*n* = 8) and control mice (*n* = 8) after Tamoxifen induction (44.4 μm ± 5.5 μm vs 43.7 μm ± 6.1 μm, *p* = 0.822). Morphometric analysis demonstrated a small but significant increase in pulmonary vascular thickness after conditional *Kdr* knockout, which was more exaggerated after chronic hypoxic exposure (Fig. 2a). α-SMA-positive area by TissueFAXS analysis after hypoxic exposure was increased in *Kdr*^*∆end*^ mice (*n* = 8) (0.55% ± 0.32% compared with 0.29% ± 0.17 in control littermates (*n* = 8), *p* < 0.05) (Fig. 2b).

Qualitative analysis of α-SMA staining after tamoxifen injection revealed no pulmonary vascular alterations in control mice under normoxia (Fig. 2c), with a mild to moderate increase in medial wall thickness after hypoxic exposure (Fig. 2d). Lungs of hypoxia-exposed *Kdr*^*∆end*^ mice revealed severe vascular remodeling with medial hypertrophy and neointimal thickening (Fig. 2e, f).

### Conditional Kdr knockout leads to obstructive vasculopathy

**Fig. 3:**
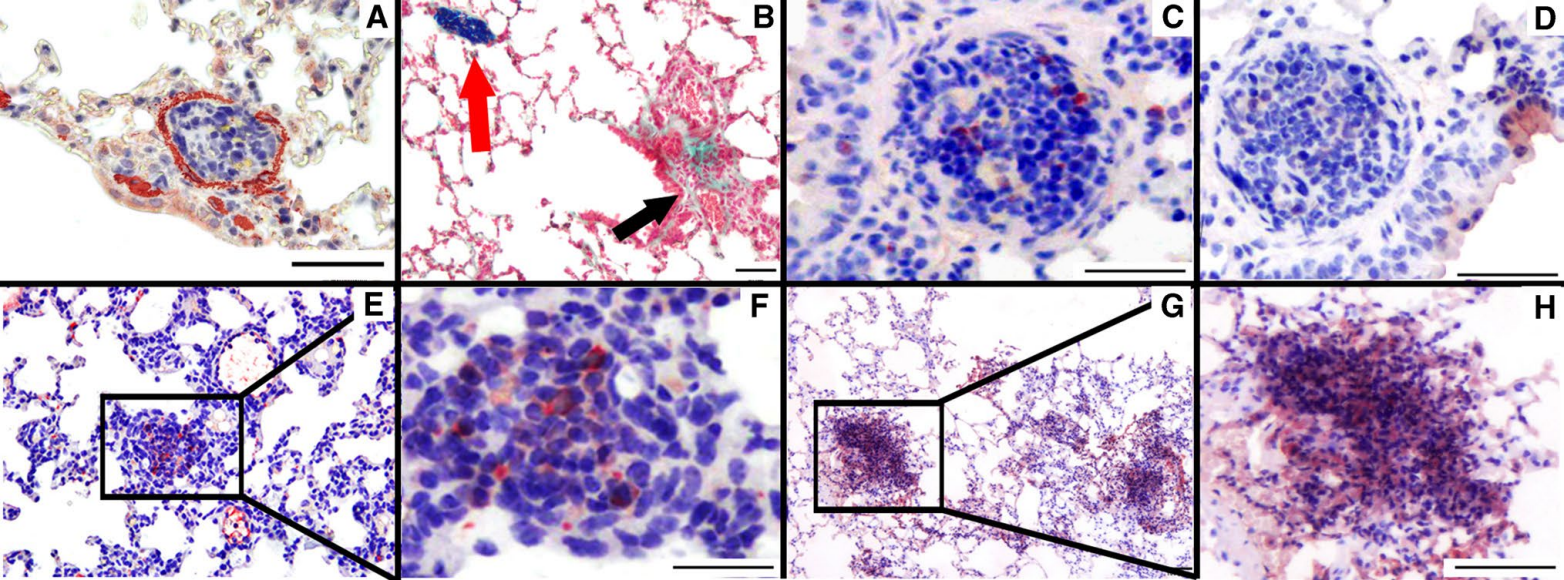
Characterization of pulmonary vascular occlusions. Vessel occlusions with proliferating cells in *Kdr*^*∆end*^ mice (**a**) (α-SMC immunostaining). Vessel occlusions with proliferating cells correspond to arteries. Black arrows indicate obstructed vessels, while red arrows identify venules that were retrogradely filled with blue ink (modified trichrome stain) (**b**). Representative sections of angioproliferative lesions found in hypoxic *Kdr*^*∆end*^ mice stain for PCNA (**c**), but not for caspase 3 (**d**). Vascular lesions containing clustered CD31-positive cells, indicating endothelial cells (**e**, **f**), clustering cells are positive for VEGFR-3 (**g**, **h**). Scale bars indicate 50 µm

**Fig. 4:**
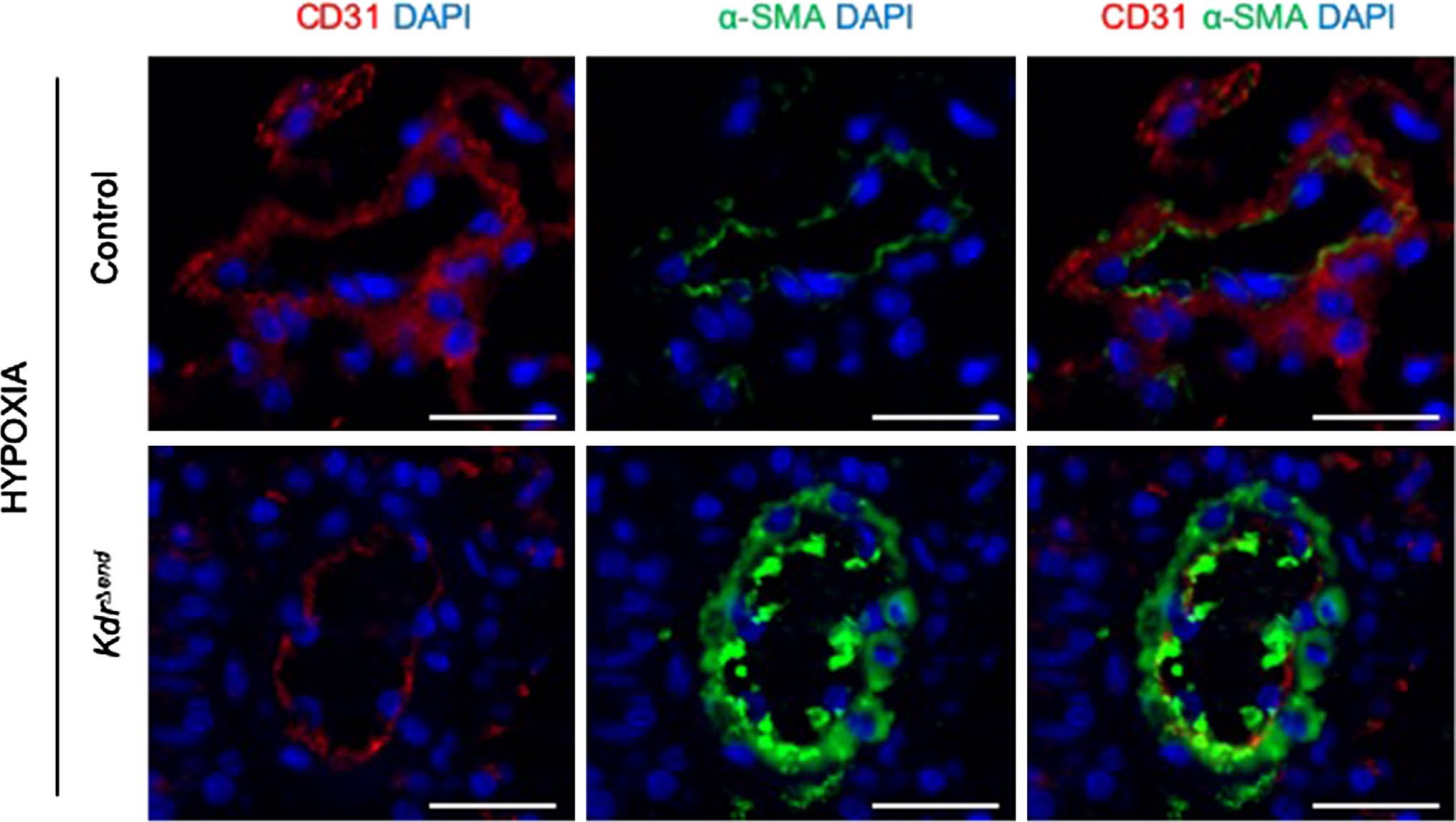
Signs of endothelial-to-mesenchymal transition after exposure to chronic hypoxia. Representative co-immunofluorescent staining of α-SMA and CD31 in lungs of control and *Kdr*^*∆end*^ mice after exposure to 6 weeks of hypoxia. Scale bars represent 20 µm

After induction and 6 weeks of hypoxic exposure of *Kdr*^*∆end*^ mice we observed various degrees of concentric medial thickening as well as vessel occlusions with proliferating cells (Fig. 3a). In a subgroup of hypoxia exposed *Kdr*^*∆end*^ mice (*n* = 4), blue ink was injected into the left ventricle to achieve retrograde filling and blue labeling of the pulmonary veins. Blue ink was only found in vessels that were free of vascular remodeling, demarcating selective remodeling of pre-capillary arterial vessels under EC-specific conditional deletion of VEGFR-2 (Fig. 3b). Thus, vessels of venous origin were not affected by the knock-out. Cells within the occlusive lesions expressed PCNA (Fig. 3c), but did not express caspase 3 (Fig. 3d) indicating the pro-proliferative nature of those occlusions. Intimal lesions were positive for CD31, suggesting an endothelial cell origin of these proliferating cells (Fig. 3e, f) and positive for VEGFR-3 (Fig. 3g, h). To further delineate the cellular composition of the neointimal thickening we performed immunofluorescent staining of α-SMA and CD31 in lungs of control and *Kdr*^*∆end*^ mice. We found decreased expression of endothelial cell marker CD31 and increased expression of mesenchymal marker α-SMA, specifically in the lungs of *Kdr*^*∆end*^ mice under hypoxia, a pattern that is consistent with the presence of endothelial to mesenchymal transition (enMT) within these lesions (Fig. 4).

### Conditional Kdr knockout leads to transient apoptosis and sustained proliferation

**Fig. 5:**
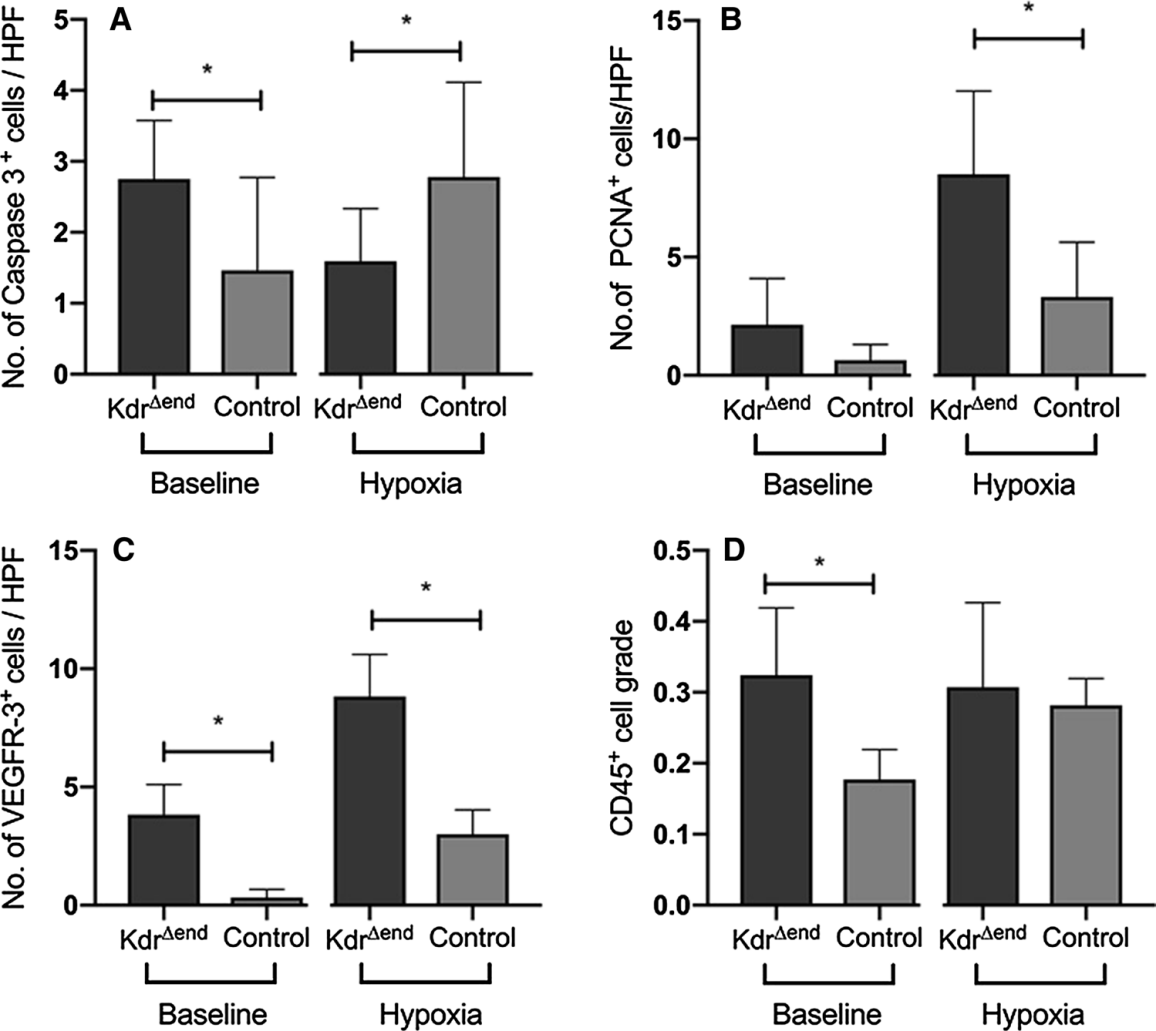
Conditional *Kdr* knockout leads to transient apoptosis sustained proliferation and vascular inflammation. Number of caspase 3-positive vascular cells in *Kdr*^*∆end*^ mice (*n* = 8) and controls (*n* = 8) at baseline and under hypoxia (**a**), number of PCNA-positive vascular cells in *Kdr*^*∆end*^ mice (*n* = 8) and controls(*n* = 8) at baseline and under hypoxia (**b**), number of VEGFR-3^+^ cells in *Kdr*^*∆end*^ mice (*n* = 6) and controls (*n* = 6) at baseline and under hypoxia (**c**), inflammatory cell grade (ratio of CD45-positive cells/total cells) in *Kdr*^*∆end*^ mice (*n* = 8) and controls (*n* = 8) at baseline and under hypoxia (**d**), Data are means ± standard deviation, statistical differences (*p < 0.05) are determined by Student’s unpaired *t* Test or Mann–Whitney *U* test as appropriate

To understand mechanisms of vascular remodeling, we examined the effects of *Kdr* knockout on apoptosis and proliferation of pulmonary vascular cells. At baseline, we observed a mild but significant increase of apoptotic cells (Fig. 5a). After hypoxic exposure, we found a small but significant increase of PCNA-positive vascular cells within remodeled arterioles (Fig. 5b), and a small decrease of caspase 3-positive vascular cells (Fig. 5a).

After conditional *Kdr* knockout we found a significant increase in VEGFR-3 positive cells in *Kdr*^*∆end*^mice (*n* = 6) as compared to control mice (*n* = 6) (3.83 cells/HPF vs. 0.33 cells/HPF, *p* = 0.024), that doubled after hypoxia (8.5 cells/HPF vs. 3.33, *p* = 0.049) (Fig. 5c) in *Kdr*^*∆end*^mice (*n* = 8) as compared to control mice (*n* = 8). We also observed inflammatory cell infiltrates in the lungs, reflected in a significant increase of the CD45-positive cell ratio in *Kdr*^*∆end*^ mice (*n* = 8). After hypoxic exposure, the inflammatory cell ratio was increased, but the combined stimulus of *Kdr* knockout and hypoxic exposure did not further increase the number of CD45-positive cells (Fig. 5d).

### Conditional Kdr knockout leads to loss of pulmonary and myocardial microvessels

**Fig. 6:**
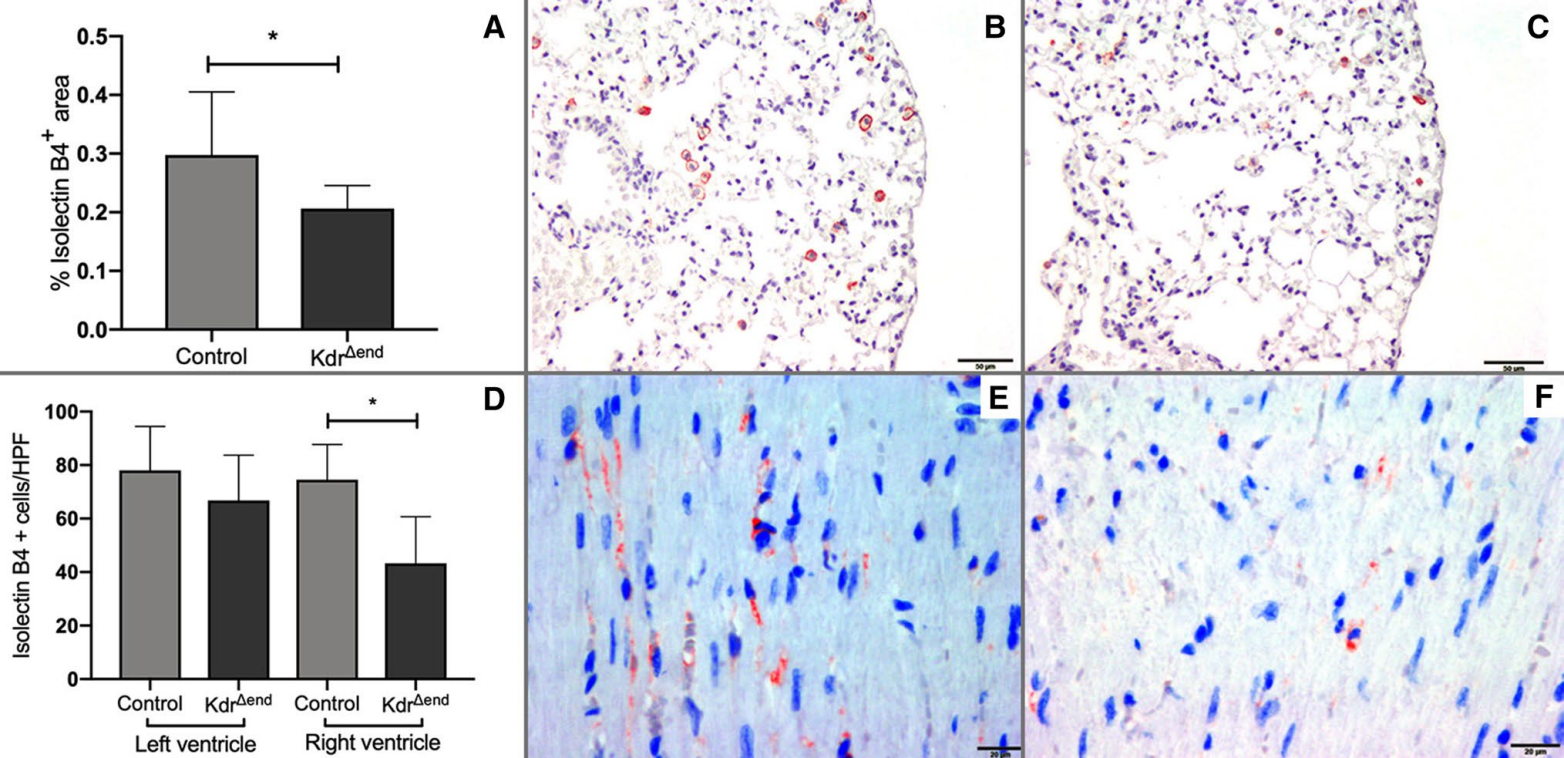
Pulmonary and myocardial microvessels of *Kdr*^*∆end*^* mice *and controls at baseline. Isolectin B4-positive area measured by TissueFAXS in pulmonary tissue (*n* = 8/group) (**a**), representative section of a pulmonary isolectin B4 stain in a control mouse (**b**) and a *Kdr*^*∆end*^ mouse (**c**), Isolectin B4-positive cells per HPF in left and right ventricle (*n* = 8/group) (**d**), representative section of a right ventricular myocardial isolectin B4 stain in a control mouse (**e**) and a *Kdr*^*∆end*^ mouse (**f**). Data are means ± SD, Statistical differences (*p < 0.05) are determined by Student’s unpaired *t* Test or Mann–Whitney *U* test as appropriate

As a consequence of *Kdr* knockout (*n* = 8), we observed a decrease of isolectin B4-positive area in lungs by TissueFAXS (0.29 ± 0.10 vs. 0.19 ± 0.04% HPF, *p* < 0.05, Fig. 6a–c). The same observation was made in the right ventricle (*n* = 8/group) (75 cells/HPF vs. 43 cells/HPF, *p* < 0.05) but not the left ventricular myocardium (*n* = 8/group) (78 cells/HPF vs 67 cells/HPF, *p* = n.s) after *Kdr* knockout (Fig. 6d–f). Isolectin B4 did not stain arterioles/arteries with > 50 µm cross sectional diameter.

### Conditional Kdr knockout leads to RV hypertrophy and failure but does not affect left ventricular function

**Fig. 7:**
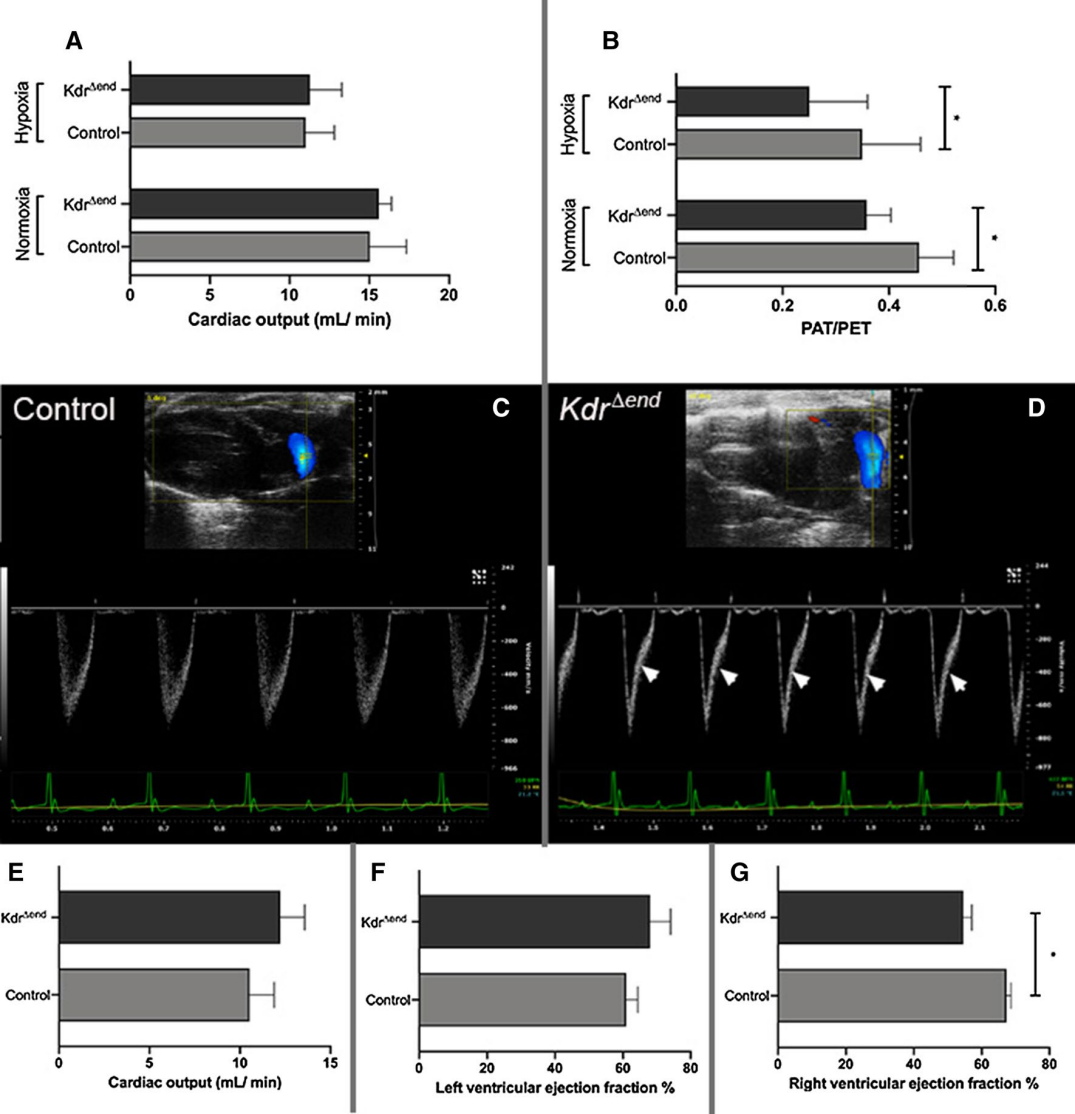
Assessment of pulmonary hypertension and ventricular function by echocardiography and cMRT. Cardiac output assessed by echocardiography (*n* = 8/group) (**a**), pulmonary hypertension depicted by decreased PA acceleration/ejection time ratio (AT/ET) (*n* = 8/group) (**b**), representative examples of pulsed Doppler from pulmonary artery (PA) flow tract recorded in parasternal long axis of control mice (**c**) and *Kdr*^*∆end*^ mice (**d**) after hypoxic exposure. White arrows indicate midsystolic notches as a sign of severe PH. Cardiac output (**e**), left ventricular (**f**) and right ventricular ejection fraction (**g**) assessed by MRT(*n* = 3/group). Data are means ± SD, Statistical differences (*p < 0.05) are determined by Student’s unpaired *t* Test

After conditional knockout, mice showed increased Fulton indices under both normoxic and hypoxic conditions (*n* = 8/group) (Fig. 1c, d). Because it has been reported, that mice exposed to the unselective VEGFR inhibitor Sugen 5416, exhibit a left heart failure phenotype [11], we investigated left and right ventricular function as well as measures of pulmonary hemodynamics using cardiac magnetic resonance tomography (MRT) (*n* = 3/group) and echocardiography (*n* = 8/group) at baseline and after hypoxic exposure. During echocardiography in anesthetized mice heart rate averaged 396 ± 28 beats per minute (bpm) and was not significantly different between *Kdr*^*∆end*^ and control mice. *Kdr*^*∆end*^ mice showed normal left ventricular function and normal cardiac output (CO) under normoxia (Fig. 7a). CO declined after hypoxic exposure, but there was no difference between study and control mice (Fig. 7a). In line with this, we found no significant differences in cardiac output and left ventricular ejection fraction as measured by cardiac MRT at baseline (Fig. 7e, f). Chronic hypoxic exposure resulted in decreased PA acceleration/ejection time ratios in both *Kdr*^*∆end*^ and control mice. These ratios were further decreased in *Kdr*^*∆end*^ mice^*.*^ (Fig. 7b). Furthermore, in the hypoxic *Kdr*^*∆end*^ mice but not in control mice we observed a midsystolic notch in the ascending slope of the PV doppler curve, a sign of severe pulmonary hypertension (Fig. 7c, d). Furthermore, there was a significant decrease of right ventricular ejection fraction by cardiac MRT in *Kdr*^*∆end*^ (*n* = 3) compared with control mice (*n* = 3) (Fig. 7g).

### Interruption of VEGF signaling leads to a significant increase in serum VEGF levels

**Fig. 8:**
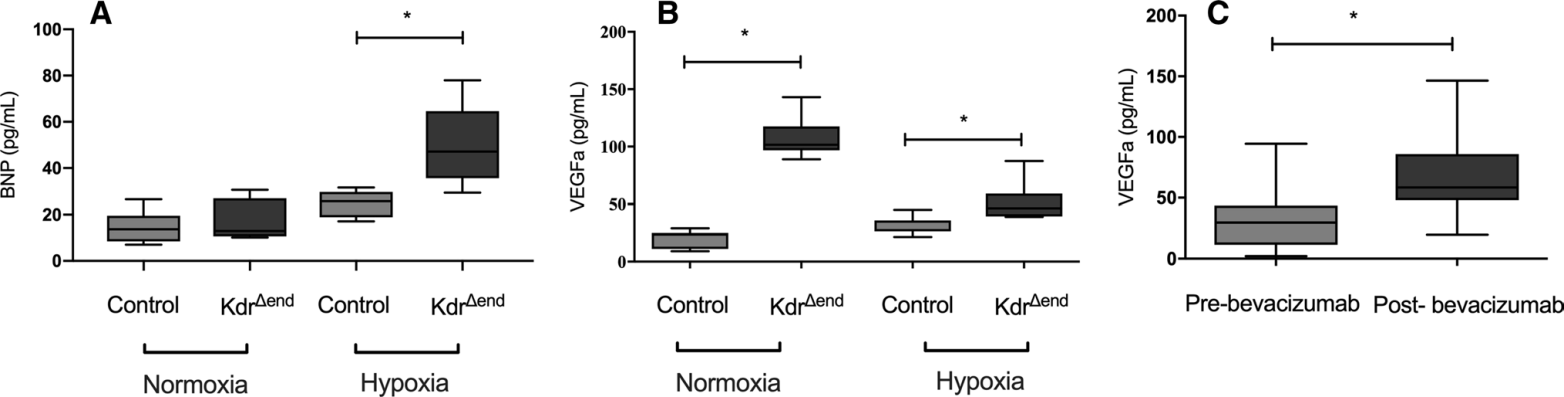
Changes in serum VEGF-a and BNP levels in response to VEGF pathway interruption. Serum BNP levels in mice at baseline and after 6 weeks of hypoxia (*n* = 8/ group) (**a**), serum VEGF-a levels of *Kdr*^*∆end*^ (*n* = 8) and control (*n* = 8) mice after induction at baseline (**b**) and after 6 weeks of exposure to hypoxia, serum VEGF-a levels in malignant meningioma patients (*n* = 34) before and on bevacizumab treatment (**c**). Statistical differences (*p < 0.05) are determined by Student’s unpaired *t* Test. Whiskers indicate1.5xIQR

To better understand the PH phenotype and the effect of *Kdr* knockout, we measured BNP and VEGFa. In *Kdr*^*∆end*^ (*n* = 8) we found a non-significant increase in BNP at baseline. After hypoxic exposure, BNP levels increased in both groups (*n* = 8/group) and were significantly higher in *Kdr*^*∆end*^ mice than in controls (49.8 ± 18.14 pg/mL vs. 25.6 ± 5.4 pg/mL, *p* < 0.05) (Fig. 8a). After *Kdr* knockout we found a significant increase of VEGFa levels in *Kdr*^*∆end*^ mice (*n* = 8) (107.3 pg/mL vs. 21.50 pg/mL in control mice (*n* = 8), *p* < 0.001). After 6 weeks of hypoxic exposure this effect was mildly attenuated, but *Kdr*^*∆end*^ mice (*n* = 8) reveled significantly higher VEGFa levels than control mice (*n* = 8) (58.00 pg/mL vs. 31.12 pg/mL, *p* = 0.005) (Fig. 8b). To investigate if this effect was reproducible in patients under anti-angiogenic therapy, we prospectively investigated baseline and on-treatment VEGFa levels in 34 patients receiving bevacizumab a monoclonal antibody directed against VEGF-a. In line with our finding in *Kdr* knockout mice we found a significant increase of serum VEGFa levels on treatment with bevacizumab compared with serum levels before treatment (68.9 ± 33.0 pg/mL vs 37.2 ± 41.5 pg/mL, *p* < 0.01) (Fig. 8c).

### Bevacizumab is associated with proliferative pulmonary vascular alterations in patients with colorectal cancer

**Fig. 9:**
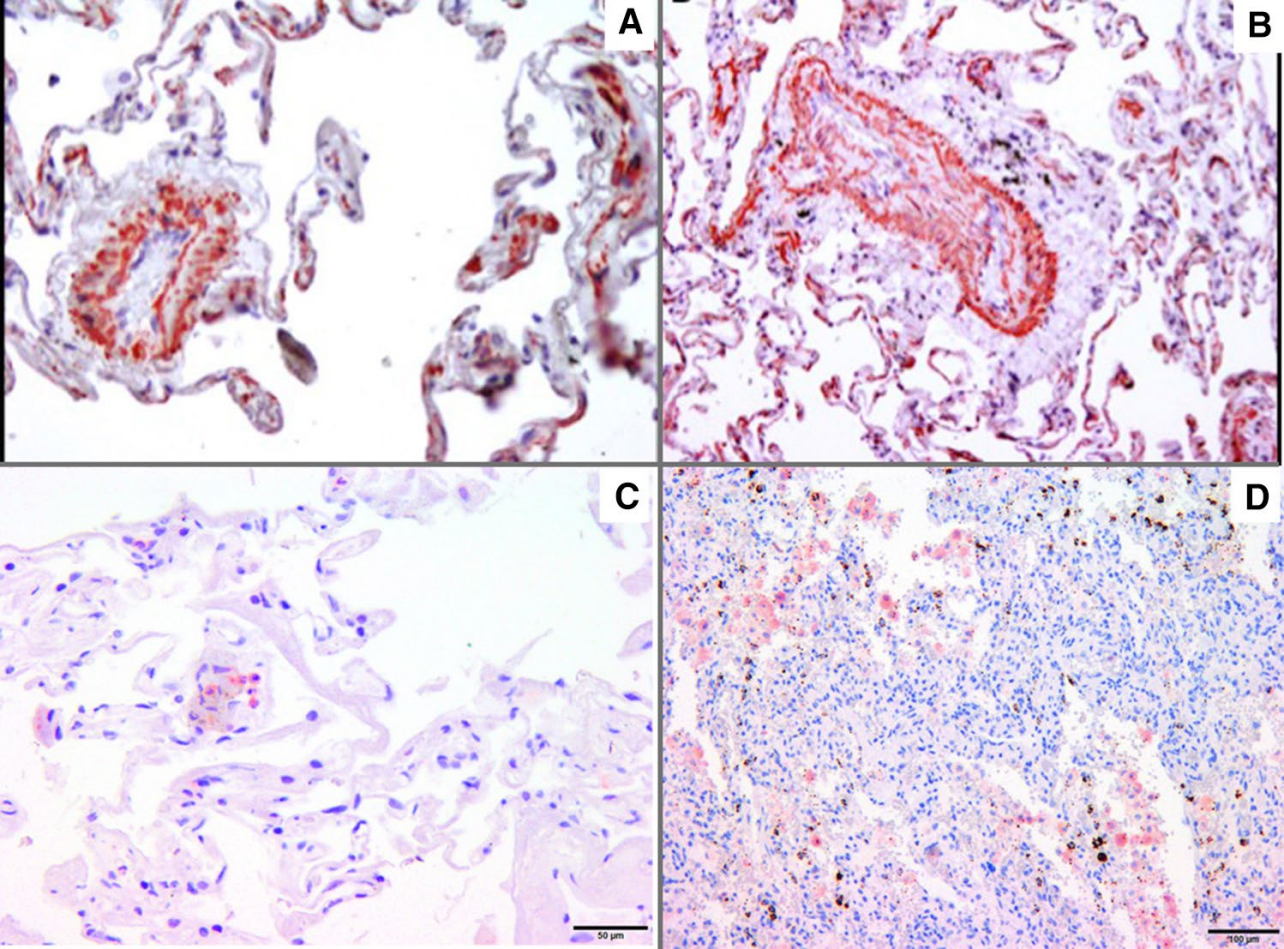
Pulmonary vascular remodeling in colorectal cancer patients on treatment with bevacizumab. Medial hypertrophy, adventitial fibrosis, semi-obstructive lesions (**a**, **b**) (α-SMC immunostaining), VEFGR-3^+^ cells obliterating vascular structures (**c**), islets of VEGFR-3^+^ cells in pulmonary tissue of bevacizumab treated patients (**d**)

To investigate the effect of anti-angiogenic therapy on the pulmonary vasculature of humans, we investigated lung samples of patients under bevacizumab therapy. Within the prospective bevacizumab registry of our Oncology department we could identify three patients who underwent pulmonary metastasectomy for colorectal cancer under treatment with bevacizumab. At the time of metastasectomy patients presented in NYHA class I or II. Computed tomography of the chest prior to metastasectomy illustrated dilated pulmonary arteries (Table 2). Throughout the histological sections various stages of vascular remodeling including medial hypertrophy, adventitial hypertrophy/fibrosis, as well as semi-occluded and totally occluded pulmonary vessels were observed (Fig. 9a, b). In all three patients we could identify either islets or vascular obstructive lesions staining positive for VEGFR-3 (Fig. 9c, d).

**Table 2:** Characteristics of patients undergoing pulmonary metastasectomy while on treatment with bevacizumab

Patient	Cancer type	Age at metastasectomy	Sex	Initial diagnosis	Chemotherapy	Bevacizumab	Symptoms	Pulmonary artery diameter	SpO_2_
1	Rectal cancer	51	Female	10.2014	8 Cycles Folfox	6 Cycles	NYHA II	32 mm	99%
2	Rectal Cancer	65	Female	11.2011	3 Cycles Folfox	3 Cycles	NYHA II	28 mm	97%
3	Rectal Cancer	42	Female	10.2015	6 Cycles Folfoxiri	6 Cycles	NYHA I	27 mm	97%

### Dysregulated expression of genes associated with Bone Morphogenetic Protein Pathway in *Kdr*^∆end^ mice

**Fig. 10:**
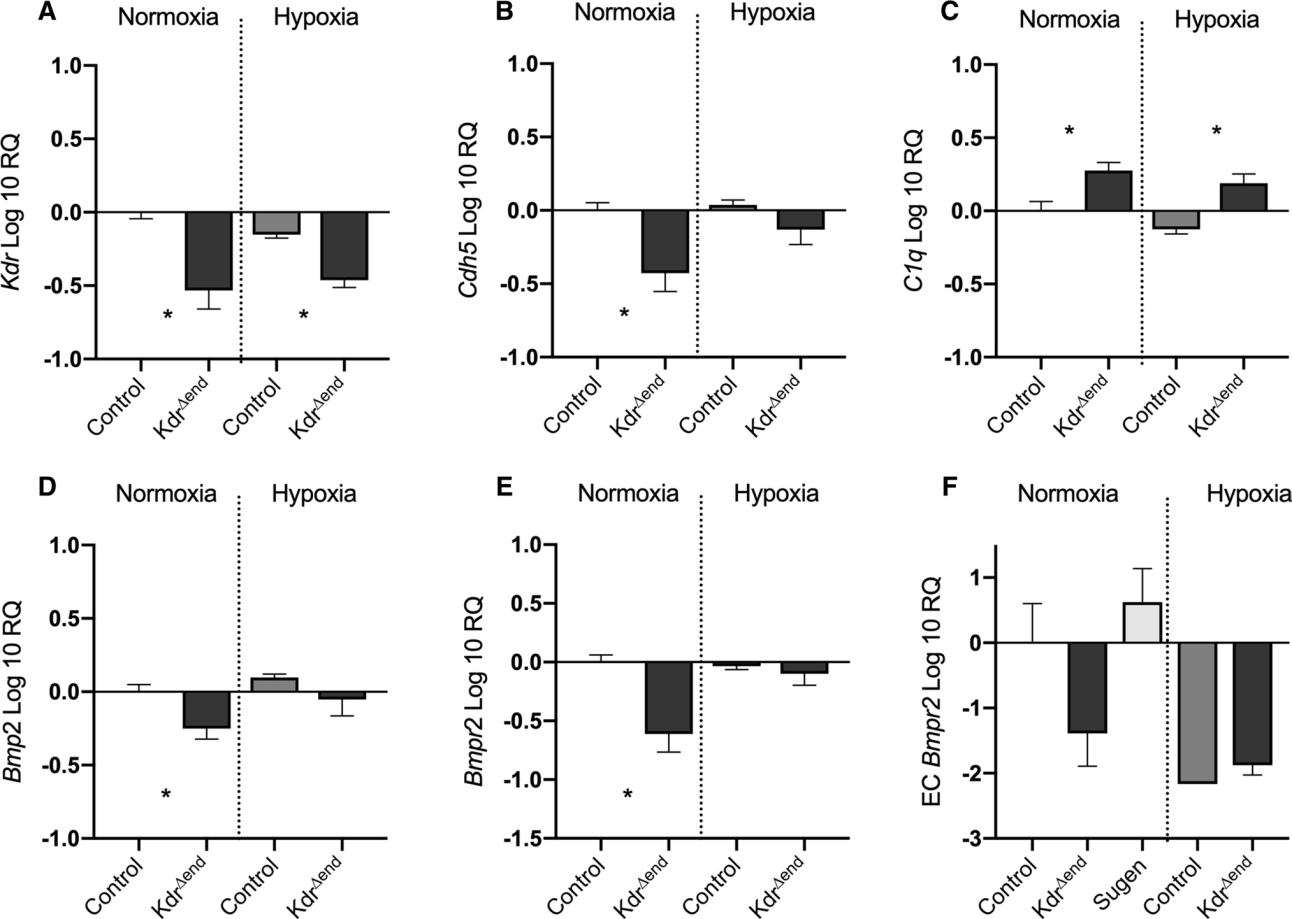
Gene expression after conditional *Kdr* deletion. Quantitative real-time polymerase chain reaction of *Kdr* (**a**), *Cdh5* (**b**), *C1q* (**c**), *Bmp2* (**d**) and *Bmpr2* (**e**), in whole lung homogenate (*n* = 8/group), pulmonary endothelial cell *Bmpr2* (*n* = 4/group) (**e**), All mRNA levels were normalized to 18S ribosomal RNA. Changes in gene expression were analyzed by ΔΔCt method. Data are means ± SD, Statistical differences (*p < 0.05) are indicated by Mann–Whitney *U* test

To further understand the link between VEGFR-2 inhibition and vascular remodeling, we investigated differential expression of bone morphogenic protein (BMP) pathway in lungs from normoxic or hypoxic *Kdr*^*∆end*^ (*n* = 8) vs. controls (*n* = 8). We confirmed downregulation of *Kdr* in *Kdr*^*∆end*^ mice that persisted after hypoxic exposure (Fig. 10a). *Kdr* knockout entailed significant down regulation of *Cdh5* mRNA (Fig. 10b). To investigate whether *Kdr* knockout leads to increased apoptosis, we investigated *C1q*, a marker of efferocytosis. We found *C1q* mRNA to be significantly upregulated after *Kdr* knockout, an effect that was attenuated under hypoxic exposure (Fig. 10c).

Interestingly *Kdr* knockout led to a significant decrease of *Bmp2*, and *Bmpr2* (Fig. 10d, e). To confirm this observation we isolated pulmonary endothelial cells after *Kdr* knockout (*n* = 4) as well as after VEGFR inhibition with SUGEN (*n* = 4) and investigated differential expression of *Bmpr2.* We found significant downregulation of *Bmpr-2* after *Kdr* knockout, but not after SUGEN treatment (Fig. 10f).

## Discussion

We investigated the effect of disrupted VEGF signaling on pulmonary vascular disease in a preclinical model of direct ablative gene manipulation of VEGFR-2. We found that endothelial cell-specific knockout in mice leads to a mild PH phenotype that is aggravated by hypoxia. Moreover we found total vessel occlusion by intimal endothelial cell proliferation and lesions consistent with enMT that resembled the pulmonary arteriopathy of human pulmonary arterial hypertension. We further hypothesized that anti-angiogenic therapies in cancer patients might cause obstructive pulmonary vascular remodeling. Therefore, we studied plasma and lung specimens from patients treated with bevacizumab, a humanized monoclonal antibody directed against VEGF. Cardiovascular side-effects of bevacizumab include thromboembolic events [22, 41], ischemic events [10, 41], hypertension [16, 28, 55], pulmonary embolism [22, 39, 41] and pulmonary hypertension [29]. The mechanism of these bevacizumab-related cardiovascular events is not fully understood. Adverse effects of VEGF inhibitors are largely consequences of blocking VEGF function in normal vascular physiology including vascular cell turnover and blood pressure regulation [21]. Preclinical evidence has shown that VEGF blockade leads to endothelial cell apoptosis in most organ systems [6]. Interestingly, this effect is reversible, resulting in vessel regrowth and normal vessel density after 1–2 weeks [31]. Histologic evaluation of lung samples obtained from pulmonary metastasectomy of patients on bevacizumab treatment showed similar vascular alterations as seen in our rodent model. We observed increased media wall thickness, perivascular fibrosis and total vessel occlusions. We assume that intimal hyperplasia may be due to selection of abnormal apoptosis-resistant endothelial cells [27, 37, 52]. Experimental proliferative pulmonary vasculopathy in a rat model was first described by Taraseviciene-Stewart who applied the VEGF receptor blocker SU5416 in combination with hypoxia [48]. More recently Ciuclan could replicate this model in mice and also observed histological changes resembling those seen in human disease [11]. Because Sugen systemically suppresses VEGFR-2 VEGFR-1, platelet-derived growth factor receptor, c-Kit (stem cell factor receptor) and RET (tyrosine kinase receptor) in all cell types and also causes emphysema [25], we selectively disrupted only VEGFR-2 signaling in endothelial cells, to dissect this pathway in PH and to overcome the pleiotropic effects on different pulmonary cells including alveolar cells type 1 and 2 [25, 36, 51]. Consequently and in contrast to the Sugen models, we did not observe severe emphysema after *Kdr* knockout. Mean linear intercept as a surrogate for alveolar enlargement was not significantly different between *Kdr*^*∆end*^ and controls after Tamoxifen induction. Therefore, we conclude that emphysema as it was observed in Sugen rat models is unlikely to depend on endothelial cell death alone. As expected [11, 48], we found that mice with disrupted VEGFR-2 signaling develop more extensive PH and RV hypertrophy than wild-type animals exposed to chronic hypoxia. In contrast to Ciuclan, but in line with Taraseviciene-Stewart, we also observed a mild PH phenotype after inhibition of VEGFR signaling without hypoxic exposure. Most importantly, we found proliferative vascular lesions expressing endothelial cell markers and VEGFR-3. There was no systemic response to *Kdr* knockout and mean systemic arterial pressure did not change in any of the treatment groups [11, 46]. However, because Ciuclan reported a left heart failure phenotype in mice following VEGFR blockade, we investigated the effect of *Kdr* knockout on LV function utilizing transthoracic echocardiography and cardiac MRT. After hypoxic exposure we observed a significant decrease of CO in all experimental groups [11], however, without further decrease by *Kdr* knockout. We used MRT to assess RV function and found significantly decreased RV ejection fraction as a consequence of *Kdr* knockout. Remarkably, *Kdr*^*∆end*^ mice show only modestly increased RV pressures at baseline while RV function was significantly impaired. We hypothesize that mechanisms other than increased RV afterload contribute to altered RV function. Bogaard has shown that isolated RV pressure overload by pulmonary artery banding leads to RV hypertrophy but not failure, whereas angioproliferative pulmonary hypertension results in both hypertrophy and RV failure. Authors hypothesized that structurally altered pulmonary circulation in PAH releases mediators that interfere with adaptive RV responses already maximally challenged to meet the increased mechanical stress [9]. Therefore, we analyzed both the pulmonary circulation and the ventricles. *Kdr* knockout leads to a loss of microvessels, more in the RV than in the LV, and in the lungs with decreased cross-sectional area of pulmonary vessels and subsequent increase in pulmonary arterial pressure [19, 34]. We hypothesize that the loss of microvessels predominantely in the RV myocardium is the sequela of the combination of *Kdr* blockade and the second ‘hit’ (chronic hypoxia and increased RV afterload) and therefore, LV myocardium remains virtually unaffected. Under hypoxia, major vessel obliterative pulmonary vascular lesions are observed in *Kdr* knockout mice that resemble intimal proliferative lesions of severe human PAH [18, 54]. To understand mechanisms of pulmonary vascular remodeling after *Kdr* knockout we examined the impact on apoptosis and proliferation of pulmonary vascular cells. Early after *Kdr* knockout we observed a small but significant increase in caspase 3-positive cells that was followed by a similar significant increase in PCNA-positive cells under hypoxia. Furthermore, we observed that the angioproliferative lesions in *Kdr*^*Δend*^ mice expressed PCNA, suggesting a proliferative phenotype. *Kdr* knockout was associated with a robust pulmonary vascular inflammatory response with accumulation of inflammatory cells in arterioles of *Kdr*^*Δend*^ mice. Because perivascular inflammatory infiltrates precede vascular remodeling in the development of PAH [40], a misguided inflammatory response to vascular injury might contribute to the development of pulmonary vasculopathy [40, 47]. However, this cellular infiltrate might also be a response to the initial vascular apoptotic processes that are superseded by angioproliferative responses. Therefore, we investigated mRNA levels of C1q, a protein that is crucial for phagocytic removal of apoptotic cells (efferocytosis). We found *C1q* mRNA to be significantly upregulated after *Kdr* knockout, which may be a signal for efferocytosis deficiency. Because VEGFR-2 has been shown to be important for macrophage–mediated efferocytosis, efferocytosis deficiency might also drive the vasculopathy observed in the present model [23, 24, 53]. We hypothesize that once efferocytosis is impaired as a consequence of *Kdr* knock-out, apoptotic cells persist and trigger inflammation and autoimmunity, leading to vascular occlusion and pulmonary hypertension [53].

In contrast to Ciuclan we found *Kdr* knockout to directly affect BMP signaling. We found both *Bmp2* and *Bmpr2* downregulated after the knockout. Although a direct relationship of VEGF and BMP signaling pathways has not been reported, their interaction seems likely. Reduced expression of *Bmp2* and *Bmpr2* suggests that both pathways act in parallel and underlines the proliferative nature of the disease resulting in a loss of patent pulmonary microvasculature, and eventually, in a loss of endothelial markers. We could identify elevated VEGFa levels as consequence of VEGFR-2 knockout or bevacizumab therapy. These findings may be central to the pathogenesis of pulmonary vasculopathy. If pulmonary hypertension is dependent on multiple injuries or “hits” [51, 52], one may speculate that we caused an initial hit via VEGF blockade and selected apoptosis resistant cells which then proliferated secondary to VEGF blockade [51]. In those proliferating cells we found a sustained upregulation of VEGFR-3, which might in part account for the pro-proliferative phenotype. VEGFR-3 shares structural similarities to VEGFR-2 and is capable to bind all members of VEGF ligands (preferentially VEGF-C and VEGF-D), promoting angiogenesis and lymphangiogenesis [3]. Because VEGFR-3 is more subjected to regulation by Notch than VEGFR-2, it may be able to rescue neoangiogenesis once VEGFR-2 is blocked [8]. We hypothesize that VEGFR-3 overexpression serves as a mechanistic explanation for the proliferative vasculopathy seen in the present model, which underpins the 2-hits-theory [51]. Thus, these data are consistent with the hypothesis that sustained VEGFR-2 inhibition in endothelial cells activates a stem cell–related cell proliferation mechanism that includes VEGFR-3 protein expression [1, 12]. Furthermore, we observed similar VEGFR-3-positive lesions in all cancer patients treated with bevacizumab. Limitations of our work are the lack of a control group for the human studies, the lack transthoracic echocardiograms and the lack of serum samples of metastasectomy patients. Not all proliferating ECs were positive for CD31 (Fig. 3g), and we could not prove monoclonal growth. Our findings in patients after bevacizumab therapy support the concept that VEGF inhibition leads to hyperproliferative endothelial cells that occlude the pulmonary vascular lumen, an observation that has been labeled as “the angiogenesis paradox in pulmonary arterial hypertension” [51]. Later, these lumenless vessels seem to disappear; however, we have no information on the mechanisms underlying the lack of EC markers in the small vessel compartments of lung and heart. Presumably, vascular changes in patients are not uniform over both lungs, but focally distributed, leading to segmental PH.

We propose that interrupted VEGF signaling leads to a pulmonary arteriopathy in rodents*.* In humans receiving anti-VEGF treatment, a similar mechanism may be effective. Our findings illustrate the importance of intact VEGF signaling for the maintenance of pulmonary vascular patency.

## Electronic supplementary material

Below is the link to the electronic supplementary material.

Supplementary file1 (DOCX 13 kb)

Supplementary file2 (JPG 572 kb)

## Abbreviations

CO: Cardiac output
EC: Endothelial cells
EnMT: Endothelial-to-mesenchymal transition
MRT: Magnetic resonance tomography
KDR: Kinase insert domain protein receptor
PAH: Pulmonary arterial hypertension
PAT: Pulmonary acceleration time
PET: Pulmonary ejection time
PH: Pulmonary hypertension
RV: Right ventricular
RVSP: Right ventricular systolic pressure
TX: Tamoxifen
VEGF: Vascular endothelial growth factor
VEGFR: Vascular endothelial growth factor receptor
