# Supplementary material for: Interruption of vascular endothelial growth factor receptor 2 signaling induces a proliferative pulmonary vasculopathy and pulmonary hypertension
Source: Basic Res Cardiol. 2020 Sep 3;115(6):58. doi: 10.1007/s00395-020-0811-5 (PMC7471204; doi:10.1007/s00395-020-0811-5)
Supplement: Supplementary file 1 — Supplementary file1 (DOCX 13 kb) [file 395_2020_811_MOESM1_ESM.docx]

**Supplemental Table 1 : Pulmonary hemodynamics**

| Right ventricular pressure | **Normoxia** |  |  | **Hypoxia** |  |  |
| --- | --- | --- | --- | --- | --- | --- |
|  | *Kdr*^∆end^ | Control | *P*-value | *Kdr*^∆end^ | Control | *P*-value |
| Baseline (mmHg) | 20.7±3.9 | 17.8±2.2 | < 0.05 | - | - | - |
| Week 2 (mmHg) | 23.8±3.4 | 19.8±2.9 | < 0.05 | 24.8±3.8 | 26.2±7.4 | n.s |
| Week 4 (mmHg) | 30.5±9.5 | 21.6±4.6 | < 0.05 | 30.2±4.7 | 25.4±5.4 | <0.05 |
| Week 6 (mmHg) | 28.5±8.9 | 19.4±2.7 | < 0.05 | 34.8±4.1 | 26.7±2.8 | <0.001 |
