# Supplementary figures and images for: Interruption of vascular endothelial growth factor receptor 2 signaling induces a proliferative pulmonary vasculopathy and pulmonary hypertension
Source: Basic Res Cardiol. 2020 Sep 3;115(6):58. doi: 10.1007/s00395-020-0811-5 (PMC7471204; doi:10.1007/s00395-020-0811-5)

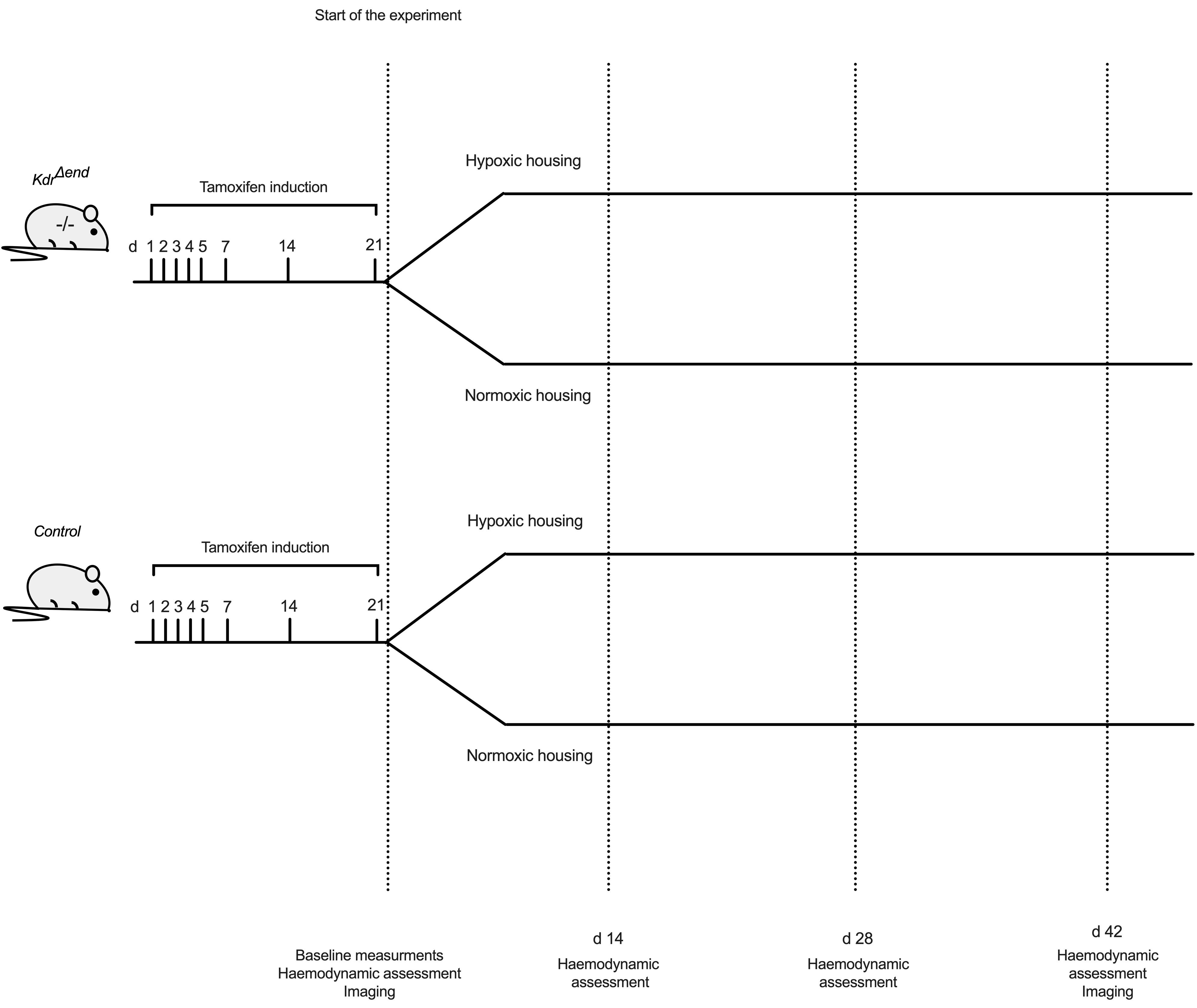

Supplement: Supplementary file 2 — Supplementary file2 (JPG 572 kb) [file 395_2020_811_MOESM2_ESM.jpg]
